# DIVA Meets EEG: Model Validation Using Formant-Shift Reflex

**DOI:** 10.3390/app13137512

**Published:** 2023-06-25

**Authors:** Jhosmary Cuadros, Lucía Z-Rivera, Christian Castro, Grace Whitaker, Mónica Otero, Alejandro Weinstein, Eduardo Martínez-Montes, Pavel Prado, Matías Zañartu

**Affiliations:** 1 Department of Electronic Engineering, Universidad Técnica Federico Santa María, Valparaíso 2390123, Chile; 2 Advanced Center for Electrical and Electronic Engineering, Universidad Técnica Federico Santa María, Valparaíso 2390123, Chile; 3 Grupo de Bioingeniería, Decanato de Investigación, Universidad Nacional Experimental del Táchira, San Cristóbal 5001, Venezuela; 4 Escuela de Ingeniería Civil Biomédica, Facultad de Ingeniería, Universidad de Valparaíso, Valparaíso 2350026, Chile; 5 Facultad de Ingeniería, Arquitectura y Diseño, Universidad San Sebastián, Santiago 8420524, Chile; 6 Centro Basal Ciencia & Vida, Universidad San Sebastián, Santiago 8580000, Chile; 7 Brain Mapping Division, Cuban Neuroscience Center, Habana 11300, Cuba; 8 Escuela de Fonoaudiología, Facultad de Odontología y Ciencias de la Rehabilitación, Universidad San Sebastián, Santiago 7510602, Chile

**Keywords:** auditory feedback, DIVA model, EEG, feedback perturbation, vocal compensation

## Abstract

The neurocomputational model ‘Directions into Velocities of Articulators’ (DIVA) was developed to account for various aspects of normal and disordered speech production and acquisition. The neural substrates of DIVA were established through functional magnetic resonance imaging (fMRI), providing physiological validation of the model. This study introduces DIVA_EEG an extension of DIVA that utilizes electroencephalography (EEG) to leverage the high temporal resolution and broad availability of EEG over fMRI. For the development of DIVA_EEG, EEG-like signals were derived from original equations describing the activity of the different DIVA maps. Synthetic EEG associated with the utterance of syllables was generated when both unperturbed and perturbed auditory feedback (first formant perturbations) were simulated. The cortical activation maps derived from synthetic EEG closely resembled those of the original DIVA model. To validate DIVA_EEG, the EEG of individuals with typical voices (N = 30) was acquired during an altered auditory feedback paradigm. The resulting empirical brain activity maps significantly overlapped with those predicted by DIVA_EEG. In conjunction with other recent model extensions, DIVA_EEG lays the foundations for constructing a complete neurocomputational framework to tackle vocal and speech disorders, which can guide model-driven personalized interventions.

## 1. Introduction

Effective oral communication is a basic and valued human daily activity [1,2]. A key aspect of this function is the sensory-motor integration for the control of speech production, which has been shown to be critical for speech acquisition [3] and that is affected in speech and voice disorders including vocal hyperfunction [4,5], stuttering and other disfluencies [6,7], as well as in neurodegenerative diseases (Parkinson’s disease) [8,9].

Studies on sensory-motor integration have traditionally used the altered auditory feedback paradigm [3], i.e., vocal compensations elicited by perturbations in the intensity, frequency, and temporality of the auditory feedback of one’s own voice.

Auditory perturbations have been studied via two approaches: (1) some trials are perturbed randomly, generating a reflexive compensatory response on the part of the participant, and (2) the perturbation is gradual, inducing the adaptation to the perturbation response. Both methods consist of recording the participant’s voice through a microphone, artificially altering speech formants or fundamental frequency, and playing back the altered vocalization to the participant in near real time through headphones [10]. Only a few studies (e.g., [11–19]) have been carried out regarding compensation in response to formant perturbation.

Research on speech production and acquisition has proposed several models of speech motor control [20]. For example, the Directions into Velocities of Articulators (DIVA) model has been developed using control theory concepts and anatomo-physiological information of brain networks. This model represents a unified neurocomputational framework that accounts for different aspects of speech production, including compensatory behaviors due to sensory feedback perturbations [21,22]. Following predictive coding [3], the DIVA model uses sensory feedback information to track and correct transient deviations from the desired vocalization. This is achieved by generating error signals that modify previously learned speech-motor programs and reconfiguring the set of motor commands associated with the activation of the articulatory and laryngeal musculature. Therefore, the DIVA model has laid the foundation for a great deal of research regarding the role of auditory feedback on speech production and acquisition in both normal-hearing and hearing-impaired populations [23–28]. Furthermore, it has become a valuable tool for assessing the etiology of stuttering, apraxia, and other speech pathologies [3,29].

The theoretical bases of the DIVA model are supported by empirical work demonstrating increased activity of the prefrontal, Rolandic and superior temporal cortices in response to auditory feedback perturbations, which has been observed using different functional modalities [30–35]. Nevertheless, the match between DIVA model predictions and experimentally acquired brain activity has been exclusively tested using functional magnetic resonance imaging (fMRI) [3,18,36]. It remains to be seen if a similar match is observed when brain activity is assessed through the electroencephalogram (EEG). It may be advantageous to the field of speech production to verify the DIVA model with EEG, as this neuroimaging modality is a direct measure of the electrical activity of the brain and allows for the representation of whole-brain oscillatory dynamics with high temporal resolution [37,38]. Furthermore, EEG is a portable, low-cost technology with relatively broad availability. Considering the large number of EEG studies assessing vocal and speech behaviors in disturbed acoustic environments [39–41], an extension of the DIVA model to EEG may contribute to disentangling key neural mechanisms of sensorimotor integration for speech-motor control.

Therefore, this study aims to investigate whether the brain activations intrinsic to DIVA match the brain activity maps estimated from EEG. To achieve this goal, the dynamics of the different DIVA maps (i.e., sets of brain nodes that collectively represent a particular type of information) [3] were obtained in three simulated conditions: (1) undisturbed auditory feedback; (2) auditory feedback with up-shifted first formant (F1); and (3) auditory feedback with down-shifted F1. The DIVA map activations corresponding to each condition were the input of a generative EEG model, which allowed for the construction of EEG scalp distributions. This extension of the DIVA model will be referred to as DIVA_EEG. Using models for solving the inverse problem in EEG, the brain cortical generators of the simulated EEG were estimated. These brain activation maps were used as a template in the experimental phase of the study, in which the event-related potentials (ERPs) elicited by each of the conditions were obtained. The cortical generators of the ERPs were estimated using source localization methods, and empirical cortical activation maps were compared with the EEG theoretical templates.

### 1.1. DIVA Model

**Figure 1.**
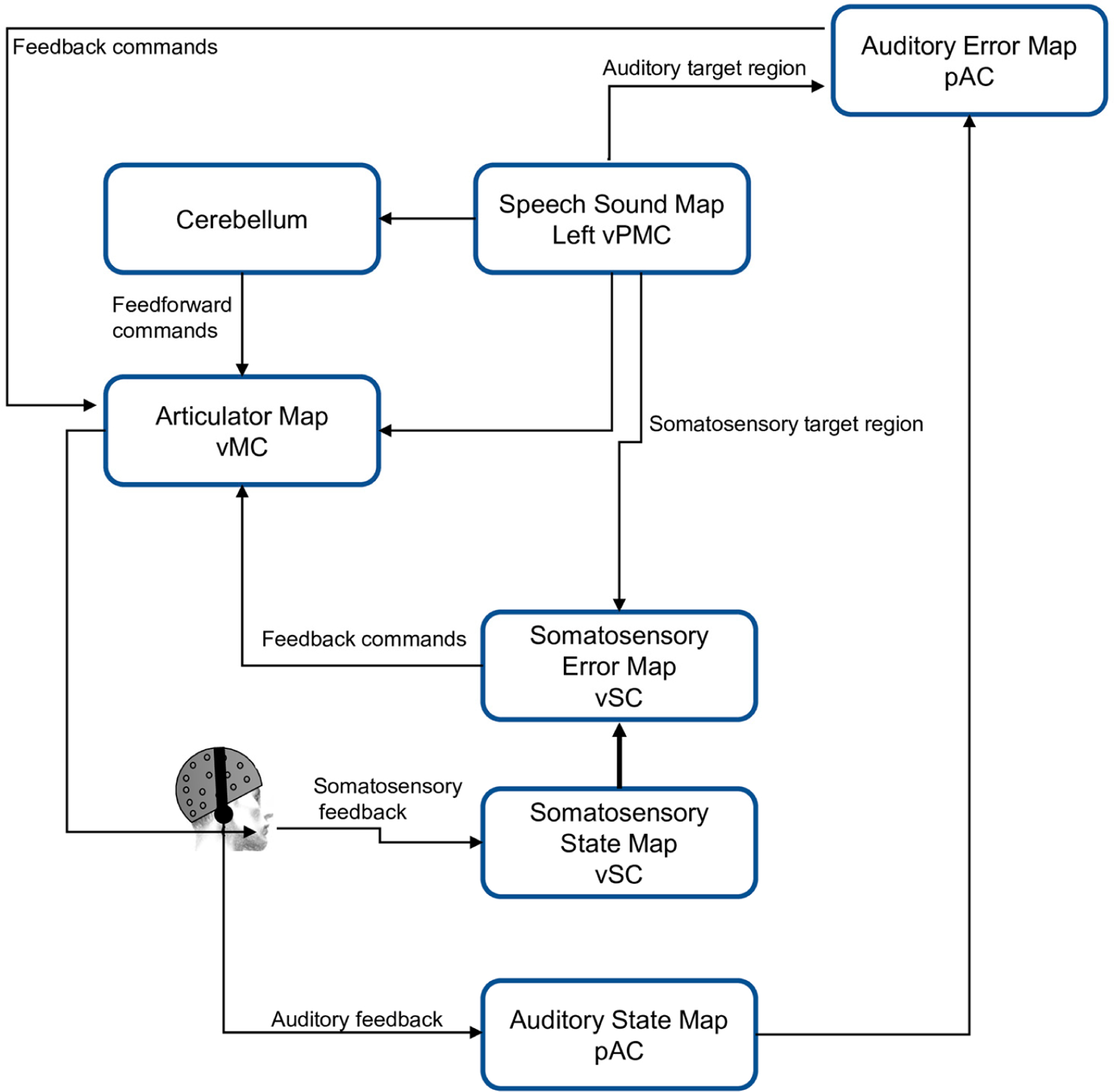
DIVA model scheme. vMC, ventral motor cortex; vPMC, ventral premotor cortex; vSC, ventral somatosensory cortex; pAC, posterior auditory cortex.

DIVA is a neurocomputational model used to simulate speech production and acquisition and it is initially designed for the English language. Each module of DIVA corresponds to a brain region activated during speech programing and production (e.g., premotor cortex, motor cortex, auditory and somatosensory cortex, cerebellum). The DIVA model is constructed as an adaptive neural network that allows for the simulation of the movement of the vocal articulators (lips, tongue, larynx, palate, and mandible) to generate speech. It also contains both a feedforward and a feedback control mechanism [3]. Figure 1 shows the structure of the model.

In the model, the production of a phoneme or syllable starts with the activation of the Speech Sound Map. Then, this information is sent to the Articulatory Velocity and Positions Maps located in the motor cortex, which control the movement of the speech articulators (vocal tract). The Auditory State Map and the Somatosensory State Map provide auditory and sensory information about how phonemes or syllables are produced. When a mismatch between the desired and actual speech production is detected, both the Auditory Error map and the Somatosensory Error Map are activated and generate a signal to correct the vocalization [3,18,36].

### 1.2. Electroencephalography (EEG)

EEG is a useful tool in clinical and research for assessing neurodevelopmental and behavioral disorders, state of consciousness, as well as in neurofeedback applications, brain–computer interfaces, among others [42–44]. The main advantage of EEG lies in its non-invasive approach for measuring the electrical activity collectively produced by large groups of neurons in the brain during information processing, with resolution in the order of milliseconds. Due to the macroscopic character of this activity and the variety of possible neural configurations responsible for a particular EEG scalp topography, it is impossible to univocally determine the EEG brain generators [45]. There are physical-mathematical algorithms that attempt to find a reasonable solution to this issue, termed the EEG inverse problem. These methods aim to estimate the brain areas responsible of the electrical potential distributions measured on the scalp [46–48].

Considering that measurements (potentials on the scalp) are only possible on a finite set of sensors and the geometric and electromagnetic characteristics of the conductive volume (head) in a discrete set of points, this relationship can be written as Equation (1), [47,48]:

(1) Φ = K ⋅ J

in which K is the matrix that expresses the linear relationship between the electric potentials on the scalp (Φ) and the average primary current density (J) at the intracerebral points.

## 2. Materials and Methods

**Figure 2.**
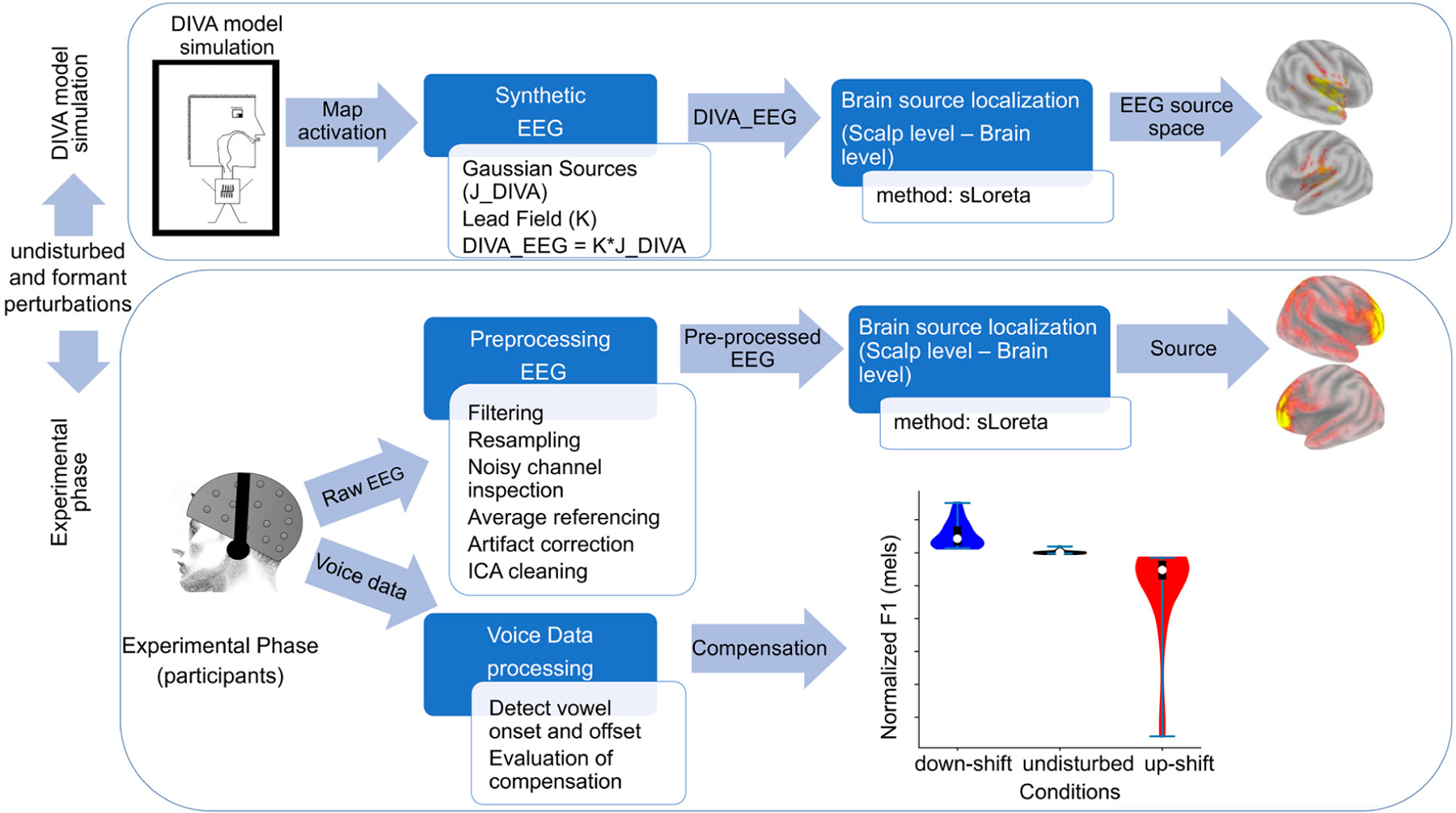
Block diagram illustrating the methodology proposed for the construction of DIVA_EEG. Both the DIVA model Simulation and the Experimental Phase of the study are presented.

The construction and the subsequent validation of DIVA_EEG consisted of two phases: DIVA model Simulation and Experimental Phase, which are illustrated in Figure 2 and described in the following subsections.

### 2.1. DIVA Model Simulation

In the present study, the main objective was to model the spatio-temporal dynamics of DIVA to obtain a template of the cortical activation associated with the DIVA observed via EEG. The outcome is the generation of EEG topographical maps that represent the activation of the different DIVA maps in each experimental condition (undisturbed, up-disturbed and down-disturbed auditory feedback).

#### 2.1.1. Simulated Speech

We chose the phoneme /e/ (defined in the model) as this vowel can readily be transformed in sounds to resemble the phoneme /æ/ (by increasing the F1 frequency) or the phoneme /i/ (by decreasing the F1 frequency). The perturbation size (F1 change in Hz) was 350 Hz. Three simulations were carried out: undisturbed, down-shift, and up-shift, under experiment type: ‘Reflexive responses’. The duration of the simulation was 550 ms, and the disturbance was applied throughout the simulation.

#### 2.1.2. Generation and Source Localization of Synthetic EEG

During simulation, the output of each DIVA node is associated with the computational load (denoted L in [3]), a term that represents the instantaneous neural activity of the node. These neural activities served as input for the EEG generative model. Therefore, point sources for the DIVA-EGG generation were seeded in brain locations that match the different nodes in the original DIVA model [3]. Table S1 shows the brain coordinates for the centroids of the seeds Traces of the synthetic EEG are displayed in Figure S1. A full-brain activity pattern was then constructed by treating the electrical activity of the seeds as Gaussian activity sources (J_DIVA) that added-up together at each brain location. The standard deviation of the normal distribution was 2. Voxels with amplitudes lower than 0.01 times the maximum amplitude were deemed inactive. The synthetic EEG (DIVA_EEG) was obtained by multiplying the simulated brain activity (J_DIVA) and the lead field K. The lead field K was computed by using a head model of three concentric, piece-wise homogeneous, and isotropic spheres [49]. Voltages (DIVA_EEG) were obtained in 64-scalp locations (a 64-electrode layout that followed the 10/20 international system for electrode placement). The DIVA-EEG is expressed by the following equation:

(2) DIVA_EEG = K ⋅ J_DIVA

where the matrix DIVA_EEG has one row for each EEG sensor and one column for each time (size Nsen × Nt), K has the number of DIVA model components as columns and is of size Nsen × Nc, and J_DIVA contains the time series of the different seeds of the model and is of (size Nc × Nt).

Brain source localizations were estimated using the standardized Low-Resolution Electromagnetic Tomography method (sLORETA, [50]; for a review, see [51]). sLORETA is based on an appropriately standardized version of the minimum norm current density estimation which overcomes problems intrinsic to the estimation of deep sources of EEG.

### 2.2. Experimental Phase

#### 2.2.1. Participants

Thirty individuals with typical voices were enrolled in this study (mean age 24 ± 3.8 years). This sample size is larger than the minimum sample necessary to conduct F-tests (repeated measure ANOVA) sensitive to large effect sizes with a statistical power of 0.8. Furthermore, the sample is sufficiently large to conduct two-tailed t-tests, able to sense large effect sizes with a statistical power of 0.8. Participants were recruited if they (1) were right-handed, (2) had no history of psychological, neurological, or speech-language disorders, (3) did not have prior training in singing, and (4) had normal binaural hearing (hearing threshold ≤ 20 dB HL at all octave frequencies between 250 and 8000 Hz). Before the experimental session, participants signed a written consent form, which was approved by the Research and Ethics Committee of the Faculty of Medicine, Universidad de Valparaíso, Chile (assessment code 52015), in compliance with the national guidelines for research with human subjects and the Declaration of Helsinki.

#### 2.2.2. Experimental Setup

This work reports reflexive responses in controls tested in an altered auditory feedback paradigm such as that utilized in [18].

Participants were seated in a comfortable chair inside a double-walled, sound-attenuating booth meeting the ANSI S3.1-1999 standard. A microphone (B&K 4961) was positioned approximately 10 cm from the participants’ mouth at a 45-degree offset in the axial direction. The acoustic signal was calibrated to physical units of dB SPL (dB re 20 μPa) using a Larson Davis calibrator (model CAL200, Depew, NY, USA).

Speech was sampled at 48 kHz using a MOTU Microbook IIc sound card and the CueMix FX software. Participants’ voices were played back to them over closed-back, over-the-ear AKG K240 Studio Headphones, with a mean latency of ~18 ms. This latency is lower than that at which feedback delays are perceived (50 ms) [52]. The speech level of the participant determined the amplitude of the speech playback.

Participants were instructed to read a series of texts presented on a screen (white font on a black background) positioned 70 cm away and adjusted in the vertical axes to the eye level of the participants at a comfortable conversational pitch and loudness. The text series comprised repetitions of the Spanish monosyllabic words: /mes/, /pep/, and /ten/. Words were presented for 2.5 s, at a presentation rate of 0.25 Hz (one word every 4 s to prevent the participants from developing a constant rhythm and the automatic character of their production). A total of 648 stimuli were presented, distributed in 6 blocks of 108 trials. In each block, stimuli were distributed in a random order. Participants were asked to sustain the vocalization of the vowel until the end of each word’s presentation. No additional instructions were provided.

A 10-trial training session was conducted prior to the start of the experiment to ensure that participants were familiar with the experimental setup, familiar with stimulus timing, and comfortable with sustaining vocalizations.

#### 2.2.3. Feedback Perturbation

**Figure 3.**
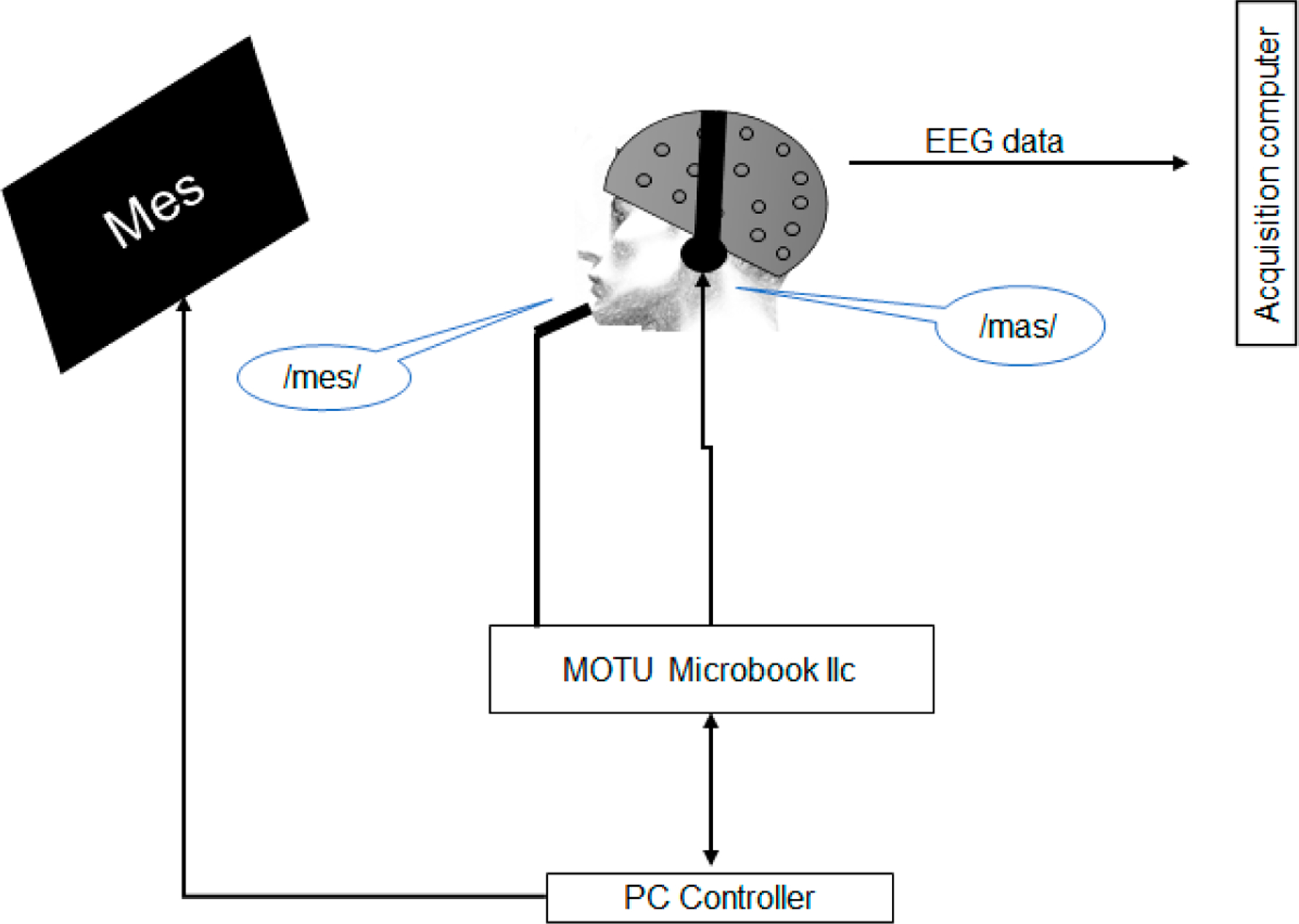
Schematic of the apparatus for applying formant perturbations. Participants produced monosyllabic words containing the vowel /e/ while their auditory feedback was perturbed toward the participant-specific vowel /a/ (e.g., participants produced /mes/ but heard a word that sounded like /mas/).

To apply the auditory perturbations, we used Audapter [29,53], a publicly available software for tracking and shifting the frequency of F1 in near real time. Both stimulus presentation and data collection were controlled by a custom MATLAB (R2022b) script (Mathworks, Natick, MA, USA) (Figure 3).

Following previous studies [18], the frequency of F1 for the auditory feedback was increased 30 percentage points relative to the produced speech signal on 1/6 of the trials (up-shift conditions: 108 trials), decreased 30 percentage points on another 1/6 of the trials (down-shift condition: 108 trial), and unaltered on the remaining 2/3 of the trials (432). After the transformation, the pronunciation of the phoneme /e/ approached either the pronunciation of the phoneme /a/ in the words /mas/, /pap/, and /tan/ (up-shifted F1), or the pronunciation of the phoneme /i/ in the words /mis/, /pip/, and /tin/ (downshifted F1) [54]. The perturbation values were different from that used in the DIVA model because the vowel triangle of the vowels in Spanish differ from that of the triangle of vowels in English)

#### 2.2.4. Processing of Acoustic Signals

Vowel onset and offset were first automatically identified with a Linear Predictive Coding model to find the frequency of F1 [55]. The compensation was evaluated in the time window between 120 and 500 ms after the vowel onset. This time window corresponds with the time at which the beginning of vocal compensations occurs [9,17,18,56,57]. Previous studies have shown that corrective responses begin between 100 and 200 milliseconds (usually 150 ms) after the onset of the perturbations and increase at least for the following 400 ms [11,15,19].

The compensatory response for each subject was calculated as follows: First, for each stimulus word the average F1 trajectory is calculated for all undisturbed trials (baseline trials). Second, the trajectory of F1 from each perturbed trial was normalized to the control condition, by subtracting the baseline from the perturbed trials. Compensatory response magnitude was calculated for each subject as the average F1 value within 120–500 ms after vowel onset [17,57].

#### 2.2.5. EEG Acquisition and Analysis

EEG was recorded using the ActiveTwo BioSemi system (BioSemi, Amsterdam, Netherlands) with ActiView acquisition software (BioSemi) with 64 scalp electrodes (10–20 electrode placement). External electrodes were placed in periocular locations to record blinks and eye movements. Analog filters were set at 0.03 and 100 Hz. During the analog/digital conversion, signals were sampled at 4096 Hz, with 24 bits of resolution. The EEG signal was pre-processed offline using standard procedures implemented in Brain Vision Analyzer 2.0^®^ (Brain Products GmbH, Munich, Germany). Recordings were re-referenced to the average of all channels and band-pass filtered between 0.1 and 40 Hz using a zero-phase shift Butterworth filter of order 8. Data were downsampled to 512 Hz. Independent Component Analysis (ICA) was used for correcting EEG artifacts induced by blinking and eye movements (following [54]). Data were segmented from −200 to 500 ms around the onset of vocalization. Semiautomatic criteria implemented in Brain Vision Analyzer were used for rejecting noisy epochs. ERPs were obtained by averaging baseline-corrected epochs. N1 and P2 peaks were identified using semiautomatic procedures. Electrodes in occipital, parietal locations and in the midline were pooled (Iz, O1, O2, Oz, P10, P7, P8, P9, PO7, PO8), and N1 and P2 amplitudes were computed as the average voltage in a two-point window around the corresponding peak amplitude. The amplitude of the N1-P2 complex was obtained and compared between conditions (unperturbed feedback, up-shifted pitch, and down-shifted pitch) using a repeated measure ANOVA (*p ≤* 0.05).

#### 2.2.6. ERP Source Localization

Brain generators of the N1-P2 complex were estimated using the standardized Low-Resolution Electromagnetic Tomography Analysis (sLORETA). For this, the 10–20 electrode layout was registered onto the scalp MNI152 coordinates. A signal-to-noise ratio of 1 was chosen for the regularization method used to compute the EEG transformation matrix (forward operator for the inverse solution problem). The standardized current density maps were obtained using a head model of three concentric spheres in a predefined source space of 6242 voxels (voxel size of 5 × 5 × 5 mm) of a reference brain (MNI 305, Brain Imaging Centre, Montreal Neurologic Institute) [58,59]. A brain segmentation of 82 anatomic compartments (cortical areas) was implemented using the automated anatomical labeling (AAL90) atlas [60].

The cortical activations (standardized current density) maps were estimated for each scalp voltage distribution in the time windows between −5 ms relative to the peak N1 amplitude and +5 ms relative to the peak P2 amplitude. Cortical activations maps obtained for the different scalp distributions were averaged. Brain cortical activity (voxel-wise activity) of the different conditions were paired-wise compared (undisturbed feedback vs. up-shifted formant, undisturbed feedback vs. down-shifted formant, and up-shifted formant vs. down-shifted formant) using two tailed t-test (α = 0.05). Results were corrected for multiple comparisons using non-parametric permutation tests (5000 randomizations) as implemented in Loreta_Key [61,62].

#### 2.2.7. Match between DIVA Related (Simulated) and ERP (Real) Cortical Activation Maps

Binarized representations of the cortical activation maps associated with feedback perturbations (maps that resulted from the statistical analyses) were obtained for both the model-driven synthetic EEG and the N1-P2 complex of the ERP (real EEG). The binarized maps were overlapped. The match between the theoretical (predicted by the model) and real (obtained from the experimental data) cortical maps was computed as a function of the number of voxels belonging to a particular AAL region that were active during the vocalization.

## 3. Results

### 3.1. DIVA Model Simulation

**Figure 4.**
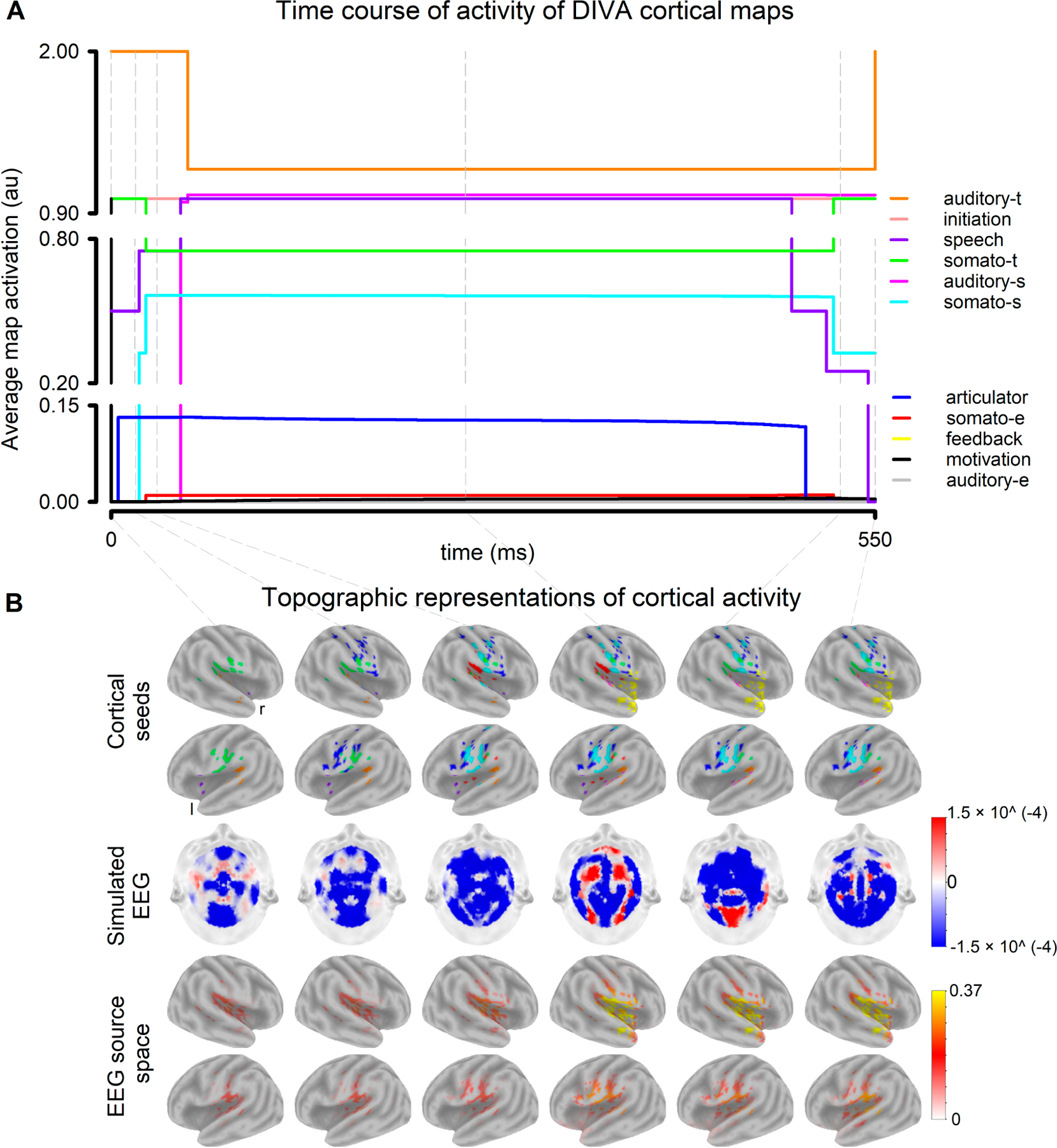
Simulations of the brain cortical activity associated with the different DIVA maps during the vocalization of the phoneme /e/ with undisturbed feedback: (**A**) Time course of activity of DIVA cortical maps. t: target, s: state, e: error (**B**) Topographic representations of cortical activity for time t = 0, 10, 25, 250, 510, 550 ms relative to the onset of the vocalization. top panel: cortical seeds. middle panel: simulated EEG. bottom panel: source space representation of the synthetic EEG.

The activation of the cortical maps of the DIVA model during the vocalization of the phoneme /e/ with undisturbed auditory feedback is illustrated in Figure 4. DIVA maps provided by the model activated at different times with respect to the onset of the simulated vocalization. The first maps were activated at t = 0 (onset of the vocalization) and were the motivation, initiation, speech, somatosensory target (somato-t) and auditory target (auditory-t) maps (Figure 4A). While the activity of the motivation map reduced to 0 directly following the vocalization onset, the activity of the initiation map remained constant (value of 1) throughout the vocalization. The articulator map (articulator) activated 10 ms after the onset of the vocalization. This was followed by the activation of the somatosensory state map (somato-s) (25 ms), the somatosensory error (somato-e) (30 ms), the feedback map (35 ms), and the auditory state (auditory-s) (55 ms after the vocalization onset). As the auditory feedback was not disturbed, the auditory error map was not activated.

**Figure 5.**
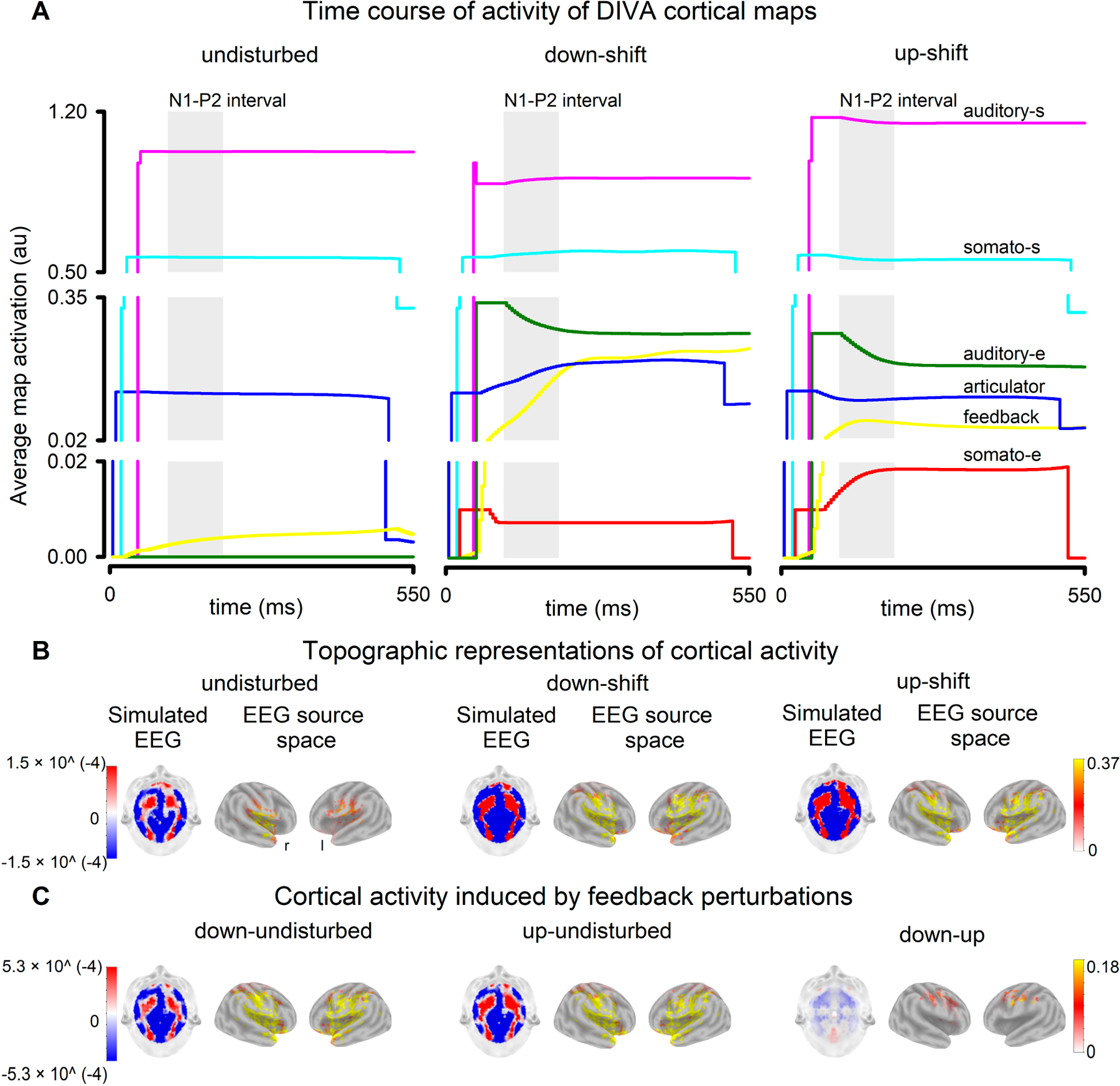
Simulations of the brain cortical activity associated with the different DIVA maps elicited by auditory feedback perturbations (F1 shifts) during the vocalization of the phoneme /e/. (**A**) Time course of activity of the DIVA cortical maps whose activity varied in response to feedback perturbations. Activities in undisturbed, downshifted, and upshifted conditions are presented. The shaded area represents the N1-P2 interval of the ERP. t: target, s: state, e: error (**B**) Scalp topography and source space representation of the synthetic EEG estimated in the time interval that corresponds to the generation of the N1-P2 complex. (**C**) Synthetic EEG (N1-P2 interval) contrasted across conditions.

Cortical activations feed into the EEG generative model, which resulted in EEG scalp distributions that characterized the different phases (stages) of the cortical dynamics (Figure 4B). Current density maps in the cerebral cortex were estimated from the EEG scalp distributions using sLORETA (Figure 4B). The EEG sources estimated with the inverse solution method closely resembled the brain distribution of DIVA maps (cortical seeds used for the EEG generation). Auditory feedback perturbations (both down- and up shift in F1) were reflected in the activity profile of the DIVA model (Figure 5A). While the activity changes of the Auditory state map clearly followed the direction of the perturbations, Somatosensory state maps changed minimally. Evident increases in the activity of the Feedback map were obtained in the presence of auditory feedback perturbation. Noteworthy, the feedback perturbation triggered the activation of both the Auditory error map and the Somatosensory error map, which are typically suppressed in undisturbed conditions.

Due to the auditory feedback perturbation, differences were observed in both the EEG scalp distributions and the activity of the EEG generators estimated with sLORETA (Figure 5B). The shifts in F1 resulted in increased bilateral activation of frontal, temporal and parietal cortical areas (Figure 5C, left and middle panels), including the orbital, opercular and triangular parts of the inferior frontal gyrus, the middle and superior frontal gyri, the Rolandic operculum, the Heschl gyrus, the temporal pole, as well as the middle and superior temporal gyri (Table S2, Supplementary Materials). The downward and upward shifts in F1, although equal in magnitude, resulted in different EEG source-space maps (Figure 5C, right panel). This asymmetry was reflected as an increase in the cortical activity elicited by down-shifted feedback perturbations in comparison with that induced by up-shifted perturbations. The differences in activity were mainly observed in frontal and parietal brain areas (bilaterally), including the primary somatosensory and motor cortices (Table S2, Supplementary Materials).

### 3.2. Behavioral and Physiological Data

**Figure 6.**
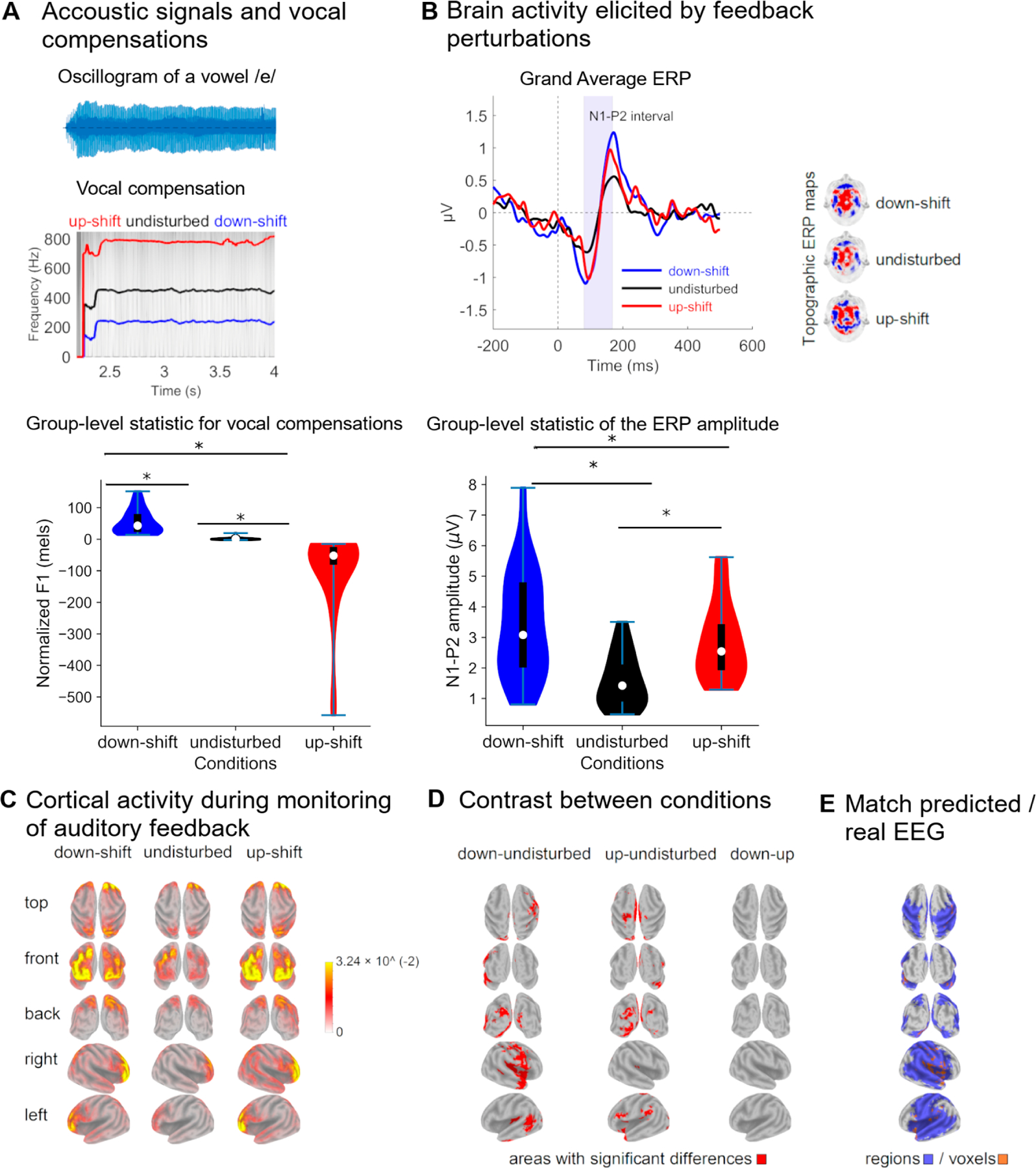
Acoustic and electrophysiological parameters describing the monitoring of one’s own vocalization. (**A**) Examples of vocal compensations elicited by F1 perturbations in the auditory feedback. In the left panel, an oscillogram representative of the phoneme /mes/ is illustrated. Likewise, the direction of the perturbation is indicated at the top of each chart. The mean F1 values of vocalizations produced in unperturbed acoustic conditions and those of vocal compensations to perturbed auditory feedback are presented in the right panel, along with the corresponding sample distributions. (**B**) Event-related potential (ERP) elicited by actively monitoring the auditory feedback of one’s own vocalizations. In the left panel, the grand average of the ERP elicited by both unperturbed and F1-shifted auditory feedback are presented. The shaded area indicates the N1-P2 complex. Scalp topography of the N1-P2 complex is illustrated in the middle panel. The mean amplitude of the N1-P2 complex elicited by unperturbed and perturbed auditory feedback are presented in the right panel, along with the corresponding sample distribution. (**C**) Current density maps illustrating the brain generators of the N1-P2 complex in the different conditions (unperturbed and perturbated auditory feedback). (**D**) Differences in the cortical activity obtained in response to unperturbed and perturbated auditory feedbacks. The difference between the current density maps elicited by F1 perturbations of equal magnitude and opposite directions is presented in the right panel. (**E**) Cortical sources of the N1-P2 complex elicited in response to F1 perturbations in the auditory feedback of one’s own vocalizations that are predicted by the DIVA model. They are illustrated both areas and voxels for which the activity predicted by the model overlapped that estimated from the real EEG. Statistically significant differences between groups are represented by *.

During the formant-shift experiment, F1 varied between conditions (F_(29,2)_ = 23.052, *p* < 0.001), as participants compensated for auditory feedback perturbations (Figure 6A, right panel). The F1 deviations counteracted the perturbational formant-shifts, such that F1 compensations were in the opposite direction to the perturbations (Figure 6A, left panel). The F1 of both types of compensations significantly differed from that of vocalizations elicited during unperturbed feedback (Holm post hoc test, *p* < 0.0.5).

F1 perturbation induced changes in the cortical activity associated with monitoring the sensory feedback of one’s own voice, which was reflected in the N1-P2 amplitude of the ERP obtained across conditions (F_(29,2)_ = 29.047, *p* < 0.001) and the changes in ERP scalp topography (Figure 6B). The N1-P2 amplitude elicited in response to both upward and downward perturbations was higher than that obtained when auditory feedback was unperturbed (Holm post hoc test, *p* < 0.001). The N1-P2 amplitude did not differ when F1 was upward and downward perturbed (Holm post hoc test, *p* = 0.36).

The cortical source of the ERP associated with monitoring of one’s own voices were estimated in large portions of the frontal, temporal, and parietal lobes (Figure 6C). It is worth noting that the activity of the N1-P2 generators significantly varied in response to F1 perturbations (*t*-test, 5000 randomizations) (Figure 6D). Downshifted F1 perturbations induced right lateralized activation of areas including the opercular, triangular and orbital parts of the inferior frontal gyrus, the Heschl gyrus (primary auditory cortex), the temporal pole, the middle and inferior temporal gyri, the Rolandic Operculum (including the primary somatosensory and motor cortices), the lingual gyrus (Figure 6D, left panel) and several sensory association cortical regions (Table S3, Supplementary Materials). Upshifted F1 perturbations resulted in a more diffuse cortical activation (Figure 6D, middle panel). Nevertheless, the cortical activations elicited by downward and upward shifts in F1 were not statistically significantly different (t-test, 5000 randomizations) (Figure 6D, right panel). Results for uncorrected comparisons are presented in Table S5, Supplementary Materials.

### 3.3. Match between DIVA Simulations and Real EEG

As upshifted and downshifted F1 perturbations did not result in statistically different cortical activations, current density maps elicited by both types of auditory feedback perturbations were merged into a single representation. This was carried out separately for activations derived from DIVA simulations (Figure 5C) and real EEG (Figure 6D), respectively. Both representations of cortical activations were binarized and contrasted to assess if cortical activity derived from DIVA simulations predicted the EEG source space of the ERP elicited by auditory feedback perturbations.

A match between the predicted and real cortical activations was obtained. This was reflected at the level of brain areas (Figure 6E left panel). Overlapping regions included the opercular part of the right inferior frontal gyrus, the Rolandic operculum (bilaterally), the temporal pole (bilaterally), the Heshl gyrus (bilaterally), the superior temporal gyrus (bilaterally), the left middle temporal gyrus, the supramarginal gyrus (bilaterally), the parietal superior gyrus (bilaterally), as well as limbic areas such as the hippocampus (bilaterally) and the insula (bilaterally) (Table 1). Overlapping was also obtained at the voxel level (Figure 6E, right panel) in frontal, temporal, parietal and limbic areas mentioned above (Table S4, Supplementary Materials).

## 4. Discussion

In this study, an extension of the DIVA model to EEG, referred to as DIVA_EEG, is presented. Neural activity of the DIVA maps associated with the vocal production and the monitoring of one’s own voice were fed into generative models of EEG. The scalp topographies of the EEG obtained in response to auditory feedback perturbations were simulated (Figures 4 and 5). Brain sources of the synthetic EEG were estimated and compared with those of the ERP (real EEG) obtained when conducting the altered auditory feedback paradigm in healthy participants (Figure 6). At the region level, a 91.5% overlapping was obtained between the model-predicted cortical activity for the control of speech production and that estimated from the experimentally acquired EEG. The overlapping between the real and predicted representations of brain activity was of 57.6% at the voxel level. Noteworthy, all the seed regions used for the EEG generative model were represented in the brain activity maps estimated from real EEG.

### 4.1. DIVA_EEG

Other modifications of the DIVA model preceded the development of DIVA_EEG. For instance, DIVA has been extended to incorporate physiologically based laryngeal motor control [63] or simplified for assessing the relative contribution of feedback and feedforward control mechanisms to sensorimotor adaptation [64]. Furthermore, DIVA has been translated to open-source codes, thereby facilitating their integration with freely available machine learning tools [65]. The DIVA environment, which also comprises the gradient order DIVA (GODIVA) for the analysis of speech sequencing [66], is now enriched with a new neuroimaging modality (EEG).

Several aspects need to be considered when interpreting the synthetic EEG that resulted from the activations of the different DIVA maps. First, DIVA_EEG comprise anatomical priors since the locations of seeds for the EEG generation are the same as for the nodes in the original DIVA model [3], which in turn were obtained from fMRI feedback perturbations protocols [18,21]. Noteworthy, since brain activity reflected in the EEG is mainly restricted to the cerebral cortex [18,36], DIVA_EEG does not include subcortical regions, which are already considered in DIVA. Second, the brain activity of DIVA_EEG seeds are simulated as Gaussian functions that extend 2mm from the centroid. Therefore, seeds in the model can be considered as a point source for the EEG generation since the seed size is lower than the voxel size of the head model used in this study for solving the EEG inverse problem [50]. Third, the main outcome of the study is presenting the first version of DIVA_EEG. The scalp topography and the cortical source of the synthetic EEG obtained with DIVA_EEG (Figures 4 and 5) are highly dependent on the head model and the theoretical considerations selected for constructing the generative EEG model and solving the EEG inverse problem. Further refinement of the DIVA_EEG can result from including individual head models [67,68], generating brain activity maps that combine the EEG obtained from DIVA_EEG and the BOLD signal obtained with DIVA [3], and testing the replicability of the results as a function of the EEG generative model [69] and the source estimation method [70]. Noteworthy, future developments can use the computational load of the nodes (the instantaneous neural activity) as input of mean field models (e.g., neural mass models) to generate oscillatory EEG-like signals for assessing the EEG oscillatory dynamic [71], including cross-frequency coupling. This aspect is relevant since accurate speech encoding has been associated with the coupling of theta oscillations that tracks slow speech fluctuations and gamma-spiking activity related to phoneme-level responses [72].

### 4.2. Vocal Compensations

Unlike the DIVA simulations, where feedback perturbations are generated by modifying the F1 of a close vowel (the English vowel /e/), the behavioral compensations of the participants were assessed by modifying an open vowel (the Spanish vowel /e/). Nevertheless, in both simulated and real perturbations, upshifts in F1 transformed the target vowel in an open vowel (/æ/ and /a/ for English and Spanish, respectively). Likewise, downshifts in F1 transformed the target vowel in a close vowel (/i/ and /i/ for English and Spanish, respectively). The vocal compensations elicited by these feedback perturbations, which typically opposes to the F1 shift (Figure 6A), replicate previous studies in which the compensatory behaviors of speakers of the target language have been assessed (e.g., Spanish [73], English [15,17,19,22] and Mandarin [74]).

Noteworthy, while compensatory behaviors typically opposed to F1 perturbations, compensations in the same direction to the F1 shift occasionally occurred (Figure S2, Supplementary Materials). This is in line with previous studies and supports the idea that, although compensations are primarily a reflex, their magnitude is modulated by several factors including attention [75], the predictability of the perturbation [1,76] and the vocal training of the participants [39,77]. Furthermore, the F1 during the compensations (Figure 6A) were closer to the F1 of the unperturbed auditory feedback than to that of the disturbed feedback, a result that has been previously reported [78,79]. Considering the interaction between different DIVA cortical maps, this has been explained by a counteracting effect of the activation of the somatosensory feedback controller on the activation of the auditory feedback controller [80].

### 4.3. ERP Elicited by Perturbations

The increased amplitude of the N1-P2 complex of the ERP elicited by auditory feedback perturbations (Figure 6B) can be considered the electrophysiological hallmark of the sensorimotor integration processes underlying the speech production [40,81,82]. The N1 component has been associated with the activation of the primary and secondary auditory cortices [83–85] and reflects the auditory processing of basic properties of acoustic stimuli. In addition, it has been suggested that P2 represents the coordinated activity of neural generators located in sensory, motor and frontal cortical regions, which might include auditory and speech-related motor areas involved in sensorimotor integration [83,86,87]. The changes in the ERP elicited by auditory feedback perturbations can be partially explained by the predictive coding models, which posits that processing of sensory information is facilitated when the sensory input is predictable [88–90]. This idea was initially proposed to explain the decreased amplitudes of N1 during active speech as compared with that obtained during the passive listening of own voices [34,83,91]. This attenuation was supposed to reflect filtering processes in which redundant information in the sensory feedback is cancelled by neural codes generated in motor-related cortical areas [92]. The hypothesis of predictability has been subsequently refined using feedback perturbations protocols [34,83,93]. Evidence shows that, the larger the differences between the expected and the incoming auditory feedback, the greater the ERP amplitude [34,83,93]. This is likely mediated by learning and reinforcing mechanisms in which predicted perturbations are segregated from the auditory re-afference, such that the disparity between the ongoing auditory feedback and the predicted feedback is reduced [1,77,83,85,94].

### 4.4. EEG Source Localization

Several methodological approaches have been used to assess the neural correlates of vocal production and control. They include, for example, the analysis of local field potentials with cortical electrodes [83] and the use of transcranial magnetic stimulation [85,95]. While these procedures enable the role of anatomically restricted brain regions to be investigated, the analysis of the whole brain activity is facilitated by methods to solve the EEG inverse problem [96,97]. The latter approach was used in this study to estimate the neural generators of the ERP elicited by self-produced speech (Figure 6C). Feedback perturbations resulted in increased activity of frontal, temporal and regions that have been traditionally associated with speech production and speech motor control (Table S3, Supplementary Materials). This group of regions include the precentral gyrus, the supplementary motor area, and the Rolandic operculum (frontal lobe), the insula (limbic lobe), the Heschl gyrus as well the inferior and superior temporal gyri (temporal lobe), and the postcentral gyrus (parietal lobe) [86].

Furthermore, differences in activity were also obtained in the occipital lobe and other limbic areas. Although this result needs to be validated, evidence suggest that speech-driven spectrotemporal receptive fields that are sensitive to pitch are located in the calcarine area, an occipital cortical region that display strong functional connections with early auditory areas [98]. Likewise, the medial and the posterior cingulate cortices have been proposed as hubs of the syllable and speech production network, respectively [99]. These networks also comprise the hippocampus, the amygdala and the insula (limbic areas), as well as the cuneus, the lingual gyrus and the inferior, middle and superior occipital gyri (occipital areas) [99].

### 4.5. Comparing Simulated and Experimentally Acquired Brain Cortical Map for Speech Motor Control

The cortical activation maps in DIVA_EEG, instead of being represented as the set of nodes obtained from DIVA, were constructed by implementing an EEG generative model to simulate EEG scalp topographies, from which current density maps in the brain were estimated. This allowed for a fair comparison between the model-based brain activity maps and those estimated from experimentally acquired EEG. An appropriate match between the predicted and the EEG-driven cortical maps was obtained, at the level of both cortical regions and voxels (Figure 6E). Differences between these cortical representations may be due to different factors, including the use of point sources for generating the synthetic EEG. Therefore, tunning the size and shape of the brain areas used as seeds for the EEG generation shall be considered for further developments of DIVA_EEG. Noteworthy, all the cortical regions selected as seeds in DIVA_EEG were present in the cortical activation maps estimated from real EEG (Table S5, Supplementary Materials). The fact that brain activation maps estimated from both synthetic and experimentally acquired EEG extends beyond the seed regions of DIVA_EEG primarily relies on the following aspects. First, the spatial resolution and precision of the EEG source estimation methods in lower than that of the fMRI. In the case of LORETA, the cortical activity is represented in a grid of 6239 voxels, each of 5×5×5 mm [50], which is much larger than the typical 1×1×1 mm voxel size of the fMRI data. Second, one of the assumptions made for solving the EEG inverse problem using LORETA is that the electrical activity of neighboring voxels has maximal similarity [100], which leads to smooth cortical activations. Third, different statistical approaches have been used for estimating speech-related cortical activation maps from fMRI [3,18,86] and EEG [95]. Finally, fMRI and EEG reflect the hemodynamic and electrical activity of the brain, respectively. In other words, these neuroimaging modalities are different in nature and have largely different dynamics. Therefore, complementary but different results are expected when assessing brain activity from EEG and fMRI. A less restricted set of cortical regions resulted from the EEG feedback perturbation paradigm (Table S4, Supplementary Materials) when compared with its analogue fMRI paradigm [3,18,86]. This indicates that speech production, rather than relying on a discrete and reduced set of brain areas, is controlled by a broadly distributed network in which information is interchanged between primary nodes (seeds in DIVA_EEG) and between them and occipital, frontal and limbic areas.

## 5. Conclusions

The extension of DIVA to include a new neuroimaging modality (EEG) will expand the use of this neurocomputational tool for assessing different aspects of speech motor control, including sensorimotor integration and predictive coding. DIVA_EEG was validated using group-level statistics of the behavior and the EEG acquired from volunteers with typical voices. Further research is needed to ascertain if the configuration parameters of DIVA_EEG can predict vocal compensatory behaviors and brain activation at individual level. Subject-specific simulations can be fostered by incorporating vocal fold control models, as carried out in LaDIVA [63], which provide a complete set of biomechanical parameters for vocal function assessment. In fact, vocal fold models associated with LaDIVA have been successfully used for subject-specific modeling of vocal hyperfunction [101]. Likewise, further extension of DIVA_EEG may consider neurophysiological muscle activation schemes for controlling vocal fold models [102] for assessing reflective and adaptive vocal behaviors at the laryngeal level. The latter may incorporate the parametrization of the sensory adaptation elicited by continuous and repetitive stimulation [103,104]. These developments are the foundations for constructing a complete and comprehensive neurocomputational framework to tackle vocal and speech disorder, which can guide model-driven personalized interventions.

## Supplementary Material

SM

## Figures and Tables

**Table 1.** Areas for which DIVA model predictions matches experimentally acquired EEG.

Brain Lobe	AAL Region	Hemisphere
	Precentral	(bilateral)
Frontal	Frontal_Inf_Oper	(right)
	Rolandic_Oper	(bilateral)
	Insula	(bilateral)
	Cingulum_Mid	(bilateral)
Limbic	Cingulum_Post	(right)
	Hippocampus	(left)
	ParaHippocampal	(bilateral)
	Heschl	(bilateral)
	Temporal_Sup	(bilateral)
Temporal	Temporal_Pole_Sup	(bilateral)
	Temporal_Mid	(bilateral)
	Temporal_Pole_Mid	(left)
	Postcentral	(bilateral)
	Parietal_Sup	(bilateral)
Parietal	Parietal_Inf	(right)
	SupraMarginal	(bilateral)
	Paracentral	(right)
	Lingual	(bilateral)
Occipital	Fusiform	(bilateral)

## Data Availability

The data presented in this study are available on request from the corresponding author to protect the personal information of participants.
