# Supplementary material for: DIVA Meets EEG: Model Validation Using Formant-Shift Reflex
Source: Appl Sci (Basel). Author manuscript; Available in PMC 2024 Mar 1. (PMC10906992; doi:10.3390/app13137512)

Supplementary Materials

Table of content **page**

Table S1: Cortical regions selected as seeds in DIVA_EEG. 2

Figure S1: Synthetic EEG (DIVA_EEG) traces, and electrode layout 4

Table S2: Numbers of active voxels in AAL90 areas in DIVA model simulations. 5

Table S3: Numbers of active voxels in AAL90 areas reflecting the by cortical activity in auditory feedback reflexive paradigms. 7

Table S4: Numbers of active voxels in AAL90 areas by cortical activity when comparing DIVA_EEG model and real EEG. 8

Figure S2: Example of compensations in the same direction of the down- shift F1. 8

Table S5: Cohen Effect Size of the cortical activations. 9

Table S1. Cortical regions selected as seeds in DIVA_EEG.

Model component	MNI_Right						MNI_Left					
	DIVA			LORETA			DIVA			LORETA		
	X	Y	Z	X	Y	Z	X	Y	Z	X	Y	Z
Motivation map	6	15	37	13	13	58	-8	13	36	-12	9	62
Initiation map	4	2	60	6	1	59	-3	-1	59	-8	-4	63
Speech sound map							-57	-1	40	-56	-2	41
Speech sound map							-55	9	0	-41	5	0
Speech sound map							-37	19	8	-46	10	8
Speech sound map	39	12	0	41	6	0	-34	13	4	-36	3	3
Feedback control map—auditory	46	23	6	45	13	6						
Feedback control map—somatosensory	41	18	-2	39	18	-10						
Feedback control map—somatosensory	53	23	6	50	14	6						
Feedback control map—somatosensory	50	8	13	52	7	6						
Articulator map—jaw	49	-10	33	53	-10	30	-51	-9	33	-51	-9	39
Articulator map—jaw	59	-3	9	62	-2	12	-59	-4	17	-56	-5	19
Articulator map—larynx	53	-3	50	55	-2	56	-48	-12	38	-47	-14	38
Articulator map—larynx	63	0	21	62	0	16	-58	-4	23	-61	-5	23
Articulator map—lip	58	-2	41	61	-2	45	-40	-17	38	-43	-16	39
Articulator map—lip	63	2	14	62	2	13	-62	4	23	-62	1	22
Articulator map—respiratory	21	-28	55	27	-28	56	-21	-28	55	-28	-30	59
Articulator map—respiratory	43	-13	38	46	-14	40	-50	-1	50	-50	-4	50
Articulator map—tongue	58	-3	29	59	-1	32	-56	-5	31	-56	-7	25
Articulator map—tongue	60	2	6	63	3	17	-61	2	13	-55	2	12
Somatosensory state map—jaw	50	-15	43	51	-17	38	-51	-11	29	-53	-15	28
Somatosensory state map—jaw	53	-13	12	53	-16	11	-53	-12	11	-51	-14	11
Somatosensory state map—larynx	49	-15	39	49	-20	40	-47	-17	35	-47	-18	36
Somatosensory state map—larynx	67	-11	12	61	-10	13	-66	-11	35	-62	-10	34
Somatosensory state map—lip	52	-10	28	51	-14	26	-62	-16	34	-62	-14	33
Somatosensory state map—lip	66	-16	18	58	-15	22	-65	-12	28	-62	-11	27
Somatosensory state map—respiratory	19	-30	56	25	-30	61	-22	-30	56	-26	-32	61
Somatosensory state map—tongue	61	-6	25	62	-6	23	-59	-9	21	-59	-10	19
Somatosensory target map	66	-16	24	61	-12	24	-65	-16	21	-57	-15	22
Somatosensory target map	60	-35	20	49	-32	20	-59	-19	30	-59	-22	30
Somatosensory error map	66	-16	24	59	-15	27	-65	-16	21	-59	-15	17
Somatosensory error map	57	-22	34	57	-22	38	-54	-25	33	-53	-25	33
Somatosensory error map	58	-31	34	56	-31	39						
Somatosensory error map	64	-41	28	55	-30	27						
Somatosensory error map	60	-35	20	52	-30	23	-60	-36	31	-57	-27	31
Somatosensory error map	60	-25	25	56	-25	27	-61	-24	23	-53	-24	22
Somatosensory error map	41	-10	-7	41	-8	-6	-39	-9	-8	-39	-9	-7
Somatosensory error map	40	-7	9	40	-14	18	-43	-11	2	-42	-12	14

Auditory state map	64	-22	-4	43	-22	-5	-65	-22	-2	-51	-18	-3
Auditory state map	58	-34	1	42	-34	7	-68	-30	7	-53	-27	11
Auditory state map	52	-28	10	52	-21	12	-51	-37	15	-47	-37	20
Auditory state map	65	-15	6	59	-14	13	-61	-12	5	-58	-13	12
Auditory state map	62	2	-3	57	1	-6	-55	1	-3	-57	3	-3
Auditory state map	49	-33	-5	38	-30	-2	-57	-42	4	-34	-41	3
Auditory state map	40	-23	3	42	-26	10	-45	-30	6	-48	-29	12
Auditory state map	61	-28	10	56	-18	11	-37	-25	3	-35	-24	9
Auditory target map	69	-30	2	56	-13	2	-68	-31	7	-53	-27	11
Auditory target map	60	-39	6	42	-34	7	-66	-38	15	-53	-28	15
Auditory target map	56	-21	-5	54	-5	-4	-56	-26	6	-53	-27	11
Auditory target map	54	-30	12	42	-30	11	-46	-39	19	-42	-40	19
Auditory error map	69	-30	2	56	-13	2	-66	-38	15	-53	-28	15
Auditory error map	60	-39	6	42	-34	7	-56	-26	6	-53	-27	11
Auditory error map	56	-21	-5	54	-5	-4	-46	-39	19	-42	-40	19
Auditory error map	54	-30	12	42	-30	11	-68	-31	7	-53	-27	11

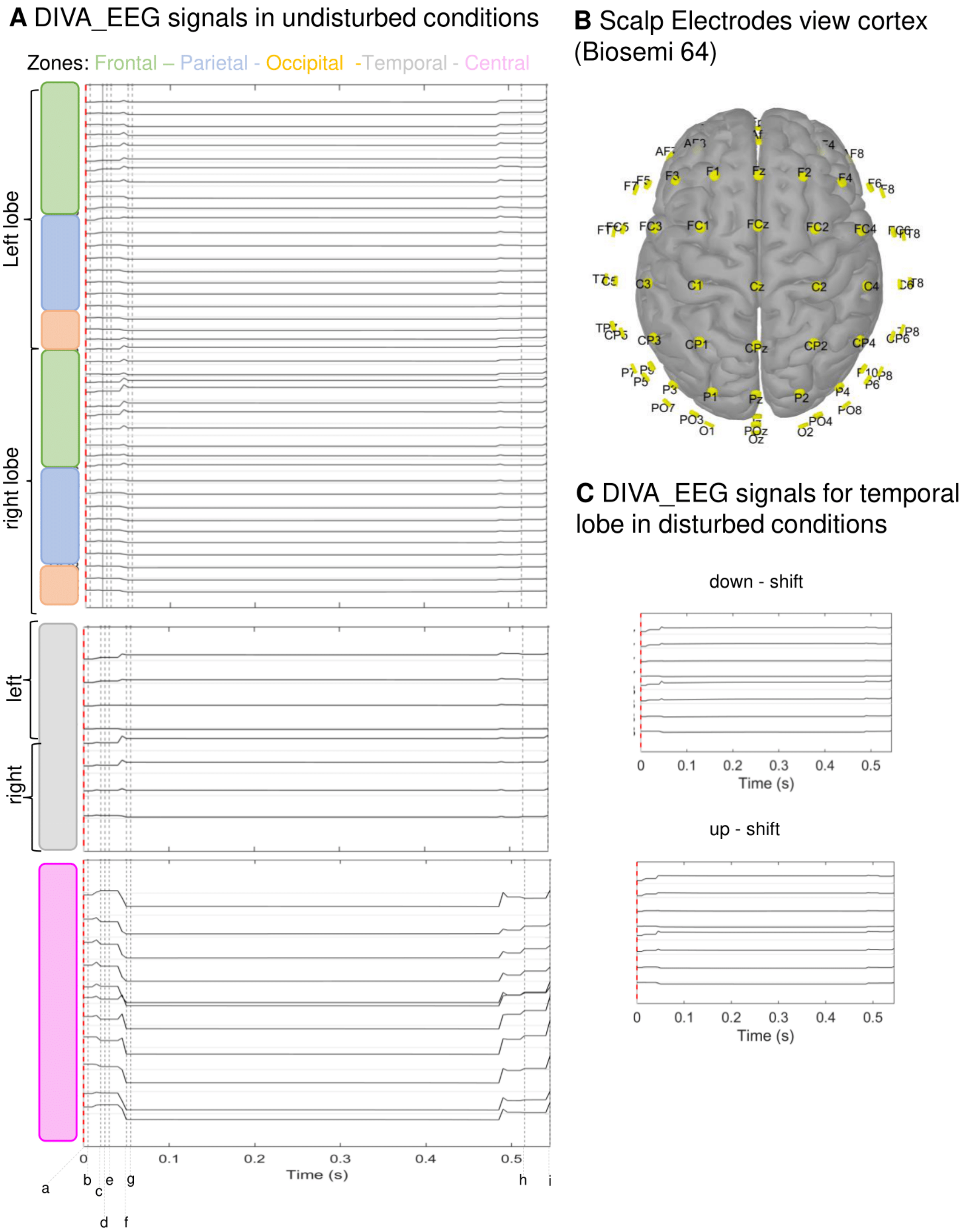

Figure S1. Synthetic EEG (DIVA_EEG) traces, montage, and map activations. (**A**). DIVA_EEG for each of the brain areas, each of the letters (in the dotted vertical lines) represents the time of activation/deactivation of the following maps: (a, time=0s) initiation, motivation, speech, somato_t, auditory_t, (b, time=0.005s) articulator, (c, time=0.02s) somato_s, (d, time=0.025s) somato_e, (e, time=0.03s) feedback, (f, time=0.05s) auditory_s, (g, time=0.055s) auditory_t, (h, time=0.51s) somato_e, somato_t, (i, time=0.05s) initiation, speech, auditory_t, arti, auditory_s, somato_s, feedback. (**B**). electrode montage using in this study. (**C**) Synthetic EEG (DIVA_EEG) traces temporal lobe by down-shift and up-shift.

Table S2. Numbers of active voxels in AAL90 areas in DIVA model simulations.

Down-Undisturbed		Up-Undisturbed		Down-Up	
Area	# voxel	Area	# voxel	Area	# voxel
Precentral_R	36	Precentral_R	32	Precentral_R	37
Frontal_Sup_R	18	Frontal_Sup_R	12	Frontal_Sup_R	1
Frontal_Sup_Orb_R	4	Frontal_Sup_Orb_R	7	Frontal_Mid_R	10
Frontal_Mid_R	7	Frontal_Mid_R	6	Frontal_Inf_Oper_R	19
Frontal_Mid_Orb_R	4	Frontal_Mid_Orb_R	4	Frontal_Inf_Tri_R	6
Frontal_Inf_Oper_R	13	Frontal_Inf_Oper_R	11	Insula_R	11
Frontal_Inf_Tri_R	1	Frontal_Inf_Tri_R	1	Postcentral_R	27
Frontal_Inf_Orb_R	34	Rolandic_Oper_R	24	Parietal_Inf_R	2
Rolandic_Oper_R	24	Frontal_Inf_Orb_R	35	SupraMarginal_R	5
Supp_Motor_Area_R	16	Supp_Motor_Area_R	10	Precentral_L	23
Frontal_Med_Orb_R	2	Olfactory_R	2	Frontal_Mid_L	3
Rectus_R	11	Frontal_Med_Orb_R	2	Parietal_Inf_L	7
Insula_R	53	Rectus_R	19	Postcentral_L	20
Cingulum_Ant_R	1	Insula_R	52	SupraMarginal_L	3
Cingulum_Mid_R	6	Cingulum_Ant_R	2		
Cingulum_Post_R	5	Cingulum_Mid_R	3		
Hippocampus_R	8	Cingulum_Post_R	4		
ParaHippocampal_R	6	Hippocampus_R	10		
Amygdala_R	1	ParaHippocampal_R	19		
Cuneus_R	1	Amygdala_R	2		
Occipital_Sup_R	2	Cuneus_R	1		
Fusiform_R	2	Lingual_R	3		
Postcentral_R	78	Occipital_Sup_R	3		
Parietal_Sup_R	16	Fusiform_R	7		
Parietal_Inf_R	20	Postcentral_R	76		
SupraMarginal_R	26	Parietal_Sup_R	17		
Angular_R	3	Parietal_Inf_R	19		
Precuneus_R	27	SupraMarginal_R	25		
Paracentral_Lobule_R	2	Angular_R	3		
Heschl_R	10	Precuneus_R	31		
Temporal_Sup_R	31	Paracentral_Lobule_R	1		
Temporal_Pole_Sup_R	29	Heschl_R	10		
Temporal_Mid_R	1	Temporal_Sup_R	31		
Temporal_Pole_Mid_R	14	Temporal_Pole_Sup_R	30		
Temporal_Inf_R	3	Temporal_Mid_R	2		
Precentral_L	27	Temporal_Pole_Mid_R	18		
Frontal_Sup_Orb_L	13	Temporal_Inf_R	3		
Frontal_Mid_L	3	Precentral_L	26		
Frontal_Mid_Orb_L	5	Frontal_Sup_Orb_L	17		
Frontal_Inf_Oper_L	12	Frontal_Mid_L	20		
Frontal_Inf_Tri_L	1	Frontal_Mid_Orb_L	5		
Frontal_Inf_Orb_L	28	Frontal_Inf_Oper_L	12		

Rolandic_Oper_L	18	Frontal_Inf_Tri_L	1
Frontal_Med_Orb_L	1	Frontal_Inf_Orb_L	29
Rectus_L	5	Rolandic_Oper_L	19
Insula_L	35	Olfactory_L	3
Cingulum_Ant_L	1	Frontal_Med_Orb_L	2
Hippocampus_L	7	Rectus_L	9
ParaHippocampal_L	7	Insula_L	35
Lingual_L	2	Cingulum_Ant_L	1
Fusiform_L	9	Cingulum_Mid_L	19
Postcentral_L	44	Cingulum_Post_L	6
Parietal_Sup_L	10	Hippocampus_L	13
Parietal_Inf_L	42	ParaHippocampal_L	15
SupraMarginal_L	14	Calcarine_L	2
Angular_L	4	Cuneus_L	2
Heschl_L	7	Lingual_L	9
Temporal_Sup_L	33	Occipital_Sup_L	1
Temporal_Pole_Sup_L	19	Occipital_Mid_L	2
Temporal_Mid_L	8	Fusiform_L	17
Temporal_Pole_Mid_L	20	Postcentral_L	56
		Parietal_Sup_L	31
		Parietal_Inf_L	38
		SupraMarginal_L	13
		Angular_L	5
		Precuneus_L	40
		Heschl_L	7
		Temporal_Sup_L	33
		Temporal_Pole_Sup_L	20
		Temporal_Mid_L	8
		Temporal_Pole_Mid_L	24
		Temporal_Inf_L	4

L: left, R: right.

Table S3. Numbers of active voxels in AAL90 areas reflecting the by cortical activity in auditory feedback reflexive paradigms.

**Down-Undisturbed**		**Up-Undisturbed**	
Area	# voxel	Area	# voxel
Precentral_R	46	Supp_Motor_Area_R	8
Frontal_Mid_R	5	Insula_R	1
Frontal_Inf_Oper_R	12	Cingulum_Mid_R	52
Rolandic_Oper_R	39	Cingulum_Post_R	2
Supp_Motor_Area_R	1	ParaHippocampal_R	1
Insula_R	36	Cuneus_R	13
Cingulum_Mid_R	2	Lingual_R	22
Calcarine_R	13	Occipital_Sup_R	7
Cuneus_R	11	Fusiform_R	14
Lingual_R	14	Precuneus_R	1
Postcentral_R	30	Paracentral_Lobule_R	10
Parietal_Sup_R	1	Heschl_R	2
SupraMarginal_R	5	Precentral_L	7
Precuneus_R	2	Frontal_Sup_L	1
Heschl_R	15	Frontal_Inf_Oper_L	2
Temporal_Sup_R	36	Frontal_Inf_Tri_L	9
Temporal_Pole_Sup_R	18	Frontal_Mid_L	2
Temporal_Mid_R	13	Frontal_Inf_Orb_L	1
Temporal_Pole_Mid_R	3	Calcarine_L	15
Temporal_Inf_R	3	Cingulum_Ant_L	6
Rolandic_Oper_L	8	Cingulum_Mid_L	98
Insula_L	1	Cingulum_Post_L	1
Cingulum_Ant_L	1	Cuneus_L	24
Cingulum_Mid_L	21	Rolandic_Oper_L	4
Hippocampus_L	4	Supp_Motor_Area_L	7
ParaHippocampal_L	10	Insula_L	16
Calcarine_L	13	ParaHippocampal_L	7
Cuneus_L	26	Lingual_L	48
Lingual_L	16	Occipital_Sup_L	24
Occipital_Sup_L	23	Occipital_Mid_L	10
Occipital_Mid_L	3	Occipital_Inf_L	7
Fusiform_L	26	Fusiform_L	43
SupraMarginal_L	1	Postcentral_L	13
Angular_L	2	Parietal_Sup_L	1
Heschl_L	6	SupraMarginal_L	28
Temporal_Sup_L	7	Precuneus_L	1
Temporal_Mid_L	36	Paracentral_Lobule_L	18
Temporal_Inf_L	24	Temporal_Inf_L	6
		Temporal_Pole_Mid_L	7
		Temporal_Pole_Sup_L	19

L: left, R: right.

Table S4. Numbers of active voxels in AAL90 areas by cortical activity when comparing DIVA_EEG model and real EEG.

Perturbed-Undisturbed			
Area	# voxel	Area	# voxel
Precentral_R	12	Precentral_L	3
Frontal_Inf_Oper_R	7	Rolandic_Oper_L	6
Rolandic_Oper_R	21	Insula_L	10
Insula_R	20	Cingulum_Mid_L	13
Cingulum_Mid_R	1	Hippocampus_L	4
Cingulum_Post_R	1	ParaHippocampal_L	4
ParaHippocampal_R	1	Fusiform_L	10
Lingual_R	3	Postcentral_L	1
Fusiform_R	1	Parietal_Sup_L	1
Postcentral_R	11	Heschl_L	1
Parietal_Sup_R	1	SupraMarginal_L	7
Parietal_Inf_R	1	Temporal_Mid_L	2
SupraMarginal_R	3	Temporal_Pole_Mid_L	4
Paracentral_Lobule_R	1	Temporal_Pole_Sup_L	8
Heschl_R	10	Temporal_Sup_L	5
Temporal_Sup_R	15		
Temporal_Pole_Sup_R	10		
Temporal_Mid_R	1		

L: left, R: right.

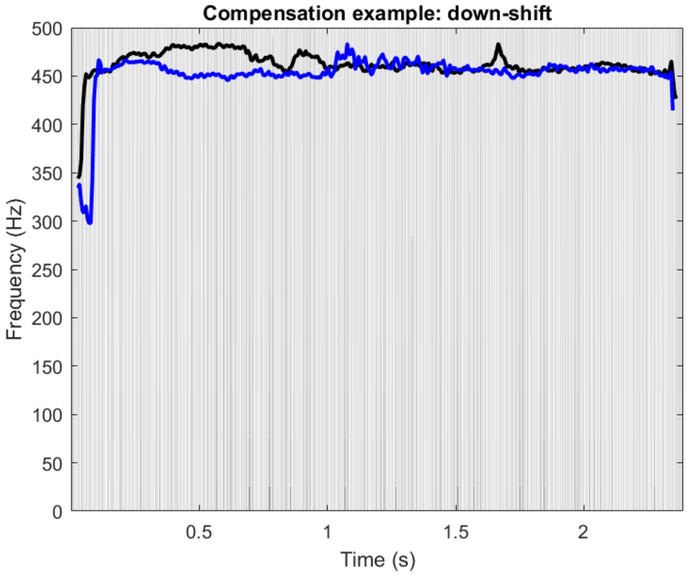

Figure S2. Example of compensations in the same direction of the down- shift F1. black line represents undisturbed and blue line represents down-shift.

Table S5. Cohen Effect Size of the cortical activations.

Small = 1.020			Medium = 2.550			Large = 4.079		
Down-undisturbed	Up-undisturbed	Down-up	Down-undisturbed	Up-undisturbed	Down-up	Down-undisturbed	Up-undisturbed	Down-up

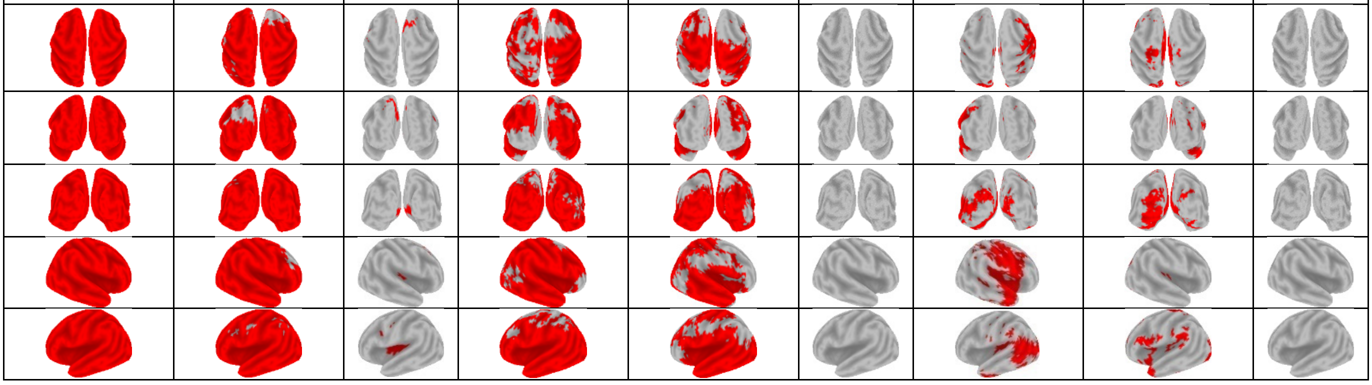

Supplement: SM [file NIHMS1959001-supplement-SM.pdf]
